# Cognitive Impairment in Relapsing Remitting and Secondary Progressive Multiple Sclerosis Patients: Efficacy of a Computerized Cognitive Screening Battery

**DOI:** 10.1155/2014/151379

**Published:** 2014-03-13

**Authors:** Athanasios Papathanasiou, Lambros Messinis, Vasileios L. Georgiou, Panagiotis Papathanasopoulos

**Affiliations:** ^1^Neuropsychology Section, Department of Neurology, University of Patras Medical School, 265 04 Patras, Greece; ^2^Department of Neurology, Essex Centre for Neurological Sciences, Queen's Hospital, Romford, RM70AG, UK; ^3^Hellenic Open University, 262 22 Patras, Greece

## Abstract

*Objective*. To investigate the pattern of cognitive impairment in relapsing remitting multiple sclerosis (RRMS) and secondary progressive multiple sclerosis (SPMS) patients using a computerized battery. *Methods*. RRMS patients (*N* = 50), SPMS patients (*N* = 30), and controls (*N* = 31) were assessed by Central Nervous System Vital Signs (CNS VS) computerized battery, Trail Making Tests (TMT) A and B, and semantic and phonological verbal fluency tasks. *Results*. The overall prevalence of cognitive dysfunction was 53.75% (RRMS 38%, SPMS 80%). RRMS patients differed from controls with large effect size on reaction time, medium effect size on TMT A and small on TMT B, phonological verbal fluency, composite memory, psychomotor speed, and cognitive flexibility. SPMS patients differed from controls in all neuropsychological measures (except complex attention) with large effect sizes on TMT A and B, phonological verbal fluency, composite memory, psychomotor speed, reaction time, and cognitive flexibility. Between patient groups, medium effect sizes were present on TMT B and psychomotor speed, while small effect sizes were present on composite memory and processing speed. *Conclusion*. CNS VS is sensitive in detecting cognitive impairment in RRMS and SPMS patients. Significant impairment in episodic memory, executive function, and processing speed were identified, with gradual increment of the frequency as disease progresses.

## 1. Introduction

The functional consequences of cognitive impairment in multiple sclerosis (MS) patients can be devastating. Cognitive impairment has a direct impact on health-related quality of life at all stages of MS [1]. It reduces physical independence and social activities [2], competence in daily activities [3], personal and community independence [4], medication adherence [5], rehabilitation potential [6], and driving safety [7]. Cognitive impaired MS patients are more likely to be unemployed, while employed MS patients are cognitive preserved [8].

Large studies of MS patients have reported cognitive impairment prevalence rates between 40 and 70% [9, 10]. Cognitive impairment has been demonstrated at all stages and in all subtypes of the disease: clinically isolated syndrome (CIS), relapsing remitting multiple sclerosis (RRMS), secondary progressive multiple sclerosis (SPMS), primary progressive multiple sclerosis (PPMS), and even benign multiple sclerosis [11]. However, the more severe levels of cognitive impairment tend to occur in the progressive phase [12]. Although almost all types of cognitive deficits can be observed in MS [13], the typical profile is information processing speed, memory, and executive function impairment, with relative preservation of language [9]. Cognitive status is typically only loosely related to disease duration [14] and physical disability [15], although larger studies have shown significant relations [16]. Cognition can also predict future disease progression as cognitive status at CIS stage predicts conversion to MS [17] and cognitive status at MS diagnosis predicts accumulation of physical disability [18].

Traditionally, evaluation of cognitive deficits in MS patients has relied on “paper-and-pencil” neuropsychological batteries. Brief Repeatable Battery of Neuropsychological Tests (BRB-N) and Minimal Assessment of Cognitive Function in MS (MACFIMS) are the most widely used [11]. However, dependence upon neuropsychologists to administer the tests, subjectivity in interpretation, and learning effects that complicate use for follow-up, all undermine their application in widespread clinical practice. In addition, full battery of tests can take several hours to complete, with additional time required for scoring and presentation of results. Computerized cognitive screening batteries have the potential to effectively address these limitations [19].

The aim of this study was to explore the efficacy of Central Nervous System Vital Signs (CNS VS) computerized battery in detecting cognitive dysfunction of MS patients in a district Greek population in Western Greece and try to designate differences in cognitive profile between RRMS and SPMS patients.

## 2. Methods

### 2.1. Participants

Eighty patients with MS, diagnosed according to McDonald criteria [20], were evaluated in our MS outpatient clinic, University of Patras, Greece. Patients were classified as RRMS (*N* = 50) and SPMS (*N* = 30). Patients with acute relapse during last three months, on corticosteroids or on other medications that could interfere with cognition, known learning disabilities, visual deficits, motor involvement of upper limb, major psychiatric illness, other neurological diseases, and non-Greek origin were excluded from this study. Expanded Disability Status Scale (EDSS) score [21] was also obtained from each patient.

In addition, thirty-one Greek control participants were recruited in order to obtain a sample with similar demographic characteristics to our patients. Exclusion criteria for the control sample included nonnative Greek speakers, visual deficits, learning disabilities, psychiatric or neurological disorder, history of brain injury, cardiovascular illness, medication use that could interfere with cognitive performance, and drug and alcohol consumption.

All groups were assessed with Beck Depression Inventory-Fast Scale in order to exclude major depression as a concomitant factor that could interfere with cognitive performance.

### 2.2. Neuropsychological Assessment

Neuropsychological assessment was performed with Central Nervous System Vital Signs (CNS VS), a recently developed computerized cognitive screening battery which also provides a Greek adapted version. Brief Core assessment of CNS VS contains seven venerable neuropsychological tests: verbal and visual memory, finger tapping, symbol digit coding, stroop, shifting attention, and continuous performance test. Final results are automatically computed, expressing patient's performance on specific domains such as composite memory, processing speed, psychomotor speed, executive function, reaction time, complex attention, and cognitive flexibility.

In addition, Greek versions of Trail Making Tests A and B, semantic and phonological verbal fluency tasks, and measuring executive function-information processing speed [22, 23] were also administered in order to broaden the cognitive screening.

We classified as cognitively impaired, patients who failed on at least 33% of the included measures [24]. We considered that patients had failed a particular test if they scored 1.5 standard deviation below (or above, if higher score corresponds to worse performance) the average performance of the control group.

### 2.3. Statistical Analysis

Differences between groups on clinical and demographic characteristics were analyzed using Kruskal-Wallis test for age and education, Mann-Whitney *U* test for EDSS and duration, and Pearson *χ* ^2^ test for gender and hand. Pearson correlation coefficients and Spearman rank correlation coefficients were calculated for age, education, EDSS, duration, and neuropsychological measures in all groups. Comparisons of performance between groups in cognitive tasks were investigated using ANOVA and Kruskal-Wallis test (whether or not normality assumption was met by Shapiro-Wilk test). Whenever a statistically significant difference was obtained, pairwise *t*-tests and Mann-Whitney tests using Holm correction were conducted and Hedges' *g* effect size indicator was calculated.

## 3. Results

### 3.1. Demographic and Clinical Data

RRMS patients were younger and had lower EDSS scores than SPMS. There were no significant differences in gender distribution and years of education between groups. Disease duration was longer for SPMS group, compared to RRMS (Table 1).

### 3.2. The Control Group

In the control sample, age and education did not correlate significantly with the performance in neuropsychological tests (Table 2).

### 3.3. Prevalence of Cognitive Dysfunction

We classified as cognitively impaired patients, those who failed on at least 33% of the included measures [24]. We considered that patients had failed a particular test if they scored 1.5 standard deviation below (or above, if higher score corresponds to worse performance) the performance of the control group.

The overall prevalence of cognitive dysfunction was 43/80 = 53.75%. Frequency of cognitive dysfunction observed for each group was for RRMS 19/50 patients (38.00%) and for SPMS 24/30 patients (80.00%) (Table 3).

#### 3.3.1. Sample of RRMS Patients

Disease duration and EDSS had a weak negative correlation with measures of processing speed, composite memory, and psychomotor speed. Years of education correlated negatively with TMT A, TMT B, reaction time, and complex attention. Age was negatively correlated with performance on semantic and phonological verbal fluency, cognitive flexibility, processing speed, composite memory, psychomotor speed, and executive function. On the other hand, disease duration had a positive correlation with reaction time; age was positively correlated with TMT B and reaction time measures, while education had a significant positive correlation with performance on phonological and semantic verbal fluency tasks, processing speed, cognitive flexibility, and executive function (Table 4(a)).

#### 3.3.2. Sample of SPMS Patients

Education had a weak negative correlation with performance on complex attention and weak positive correlation on phonological verbal fluency task. Disease duration was only positively correlated with complex attention, while age and EDSS did not have any significant correlations with task performance (Table 4(b)).

### 3.4. Comparison of Performance between Groups

Table 5(a) displays performance (mean scores) on all cognitive tasks in RRMS, SPMS patients, and controls.

Since there is a statistically significant difference between mean ages of RRMS and SPMS patients (Table 1), we decided to eliminate its effect by introducing mean age as a covariate when studying the neuropsychological variables in patient groups. To achieve it, we applied a regression analysis on each neuropsychological measure by age and used the residuals of each regression analysis to compare groups on each test.

The statistical comparison (ANOVA, Kruskal-Wallis test) of task performance between RRMS, SPMS, and controls showed statistically significant differences in all measures.

To compare task performance between group pairs, we conducted *t*-test or Mann-Whitney test, whether or not the normality assumption was met by the Saphiro-Wilk normality test. We also calculated Hedges' *g* as a measure of effect size, which is an unbiased estimator of Cohen's *d*. Particularly, in the normality assumption case, Hedges' *g* was calculated directly, while in the opposite case, Cliff's delta was calculated and transformed to Cohen's *d* and then to Hedges' *g*, so that all effect sizes estimate will have the same form and scale. A Hedges' *g* effect size of 0.2 to 0.3 is considered a “small” effect, around 0.5, a “medium” effect and above 0.8, a “large” effect.

Pairwise comparisons demonstrated that control group performed significantly better than SPMS group in all measures except complex attention. In addition the control group performed better than RRMS group in reaction time, TMT A and B, phonological verbal fluency task, composite memory, psychomotor speed, and cognitive flexibility (Table 5(b)).

Large effect sizes were present when SPMS patients were compared to controls on TMT A, TMT B, phonological verbal fluency, composite memory, psychomotor speed, reaction time, and cognitive flexibility. Medium effect sizes were present when the same groups were compared on semantic verbal fluency and executive function, while small effect size was present on processing speed. Comparison between RRMS patients and controls revealed large effect size on reaction time, medium effect size on TMT A and small on TMT B, phonological verbal fluency task, composite memory, psychomotor speed, and cognitive flexibility. Between patient groups (RRMS and SPMS), medium effect sizes were present on TMT B and psychomotor speed, while small effect sizes were present on composite memory and processing speed (Table 5(b)).

## 4. Discussion

The present study allowed the recognition of cognitive impairment in RRMS and SPMS patients in a district Greek population in Western Greece, using CNS VS computerized battery as well as Trail Making Tests A and B and semantic and phonological verbal fluency tasks. The overall prevalence of cognitive dysfunction in our patients was 53.75%, thus in accordance with the estimated prevalence of previous studies that was ranging from 40% up to 70% [9]. Cognitive dysfunction declined from RRMS (38%) to SRMS patients (80%), consistent with previous studies suggesting that cognitive deficits are more frequent and pronounced in chronic progressive MS and tend to worsen over time [12].

Higher percentages of impaired RRMS patients were found in TMT A and B (34%), phonological verbal fluency (30%), and in reaction time (58%) with large effect size in reaction time, medium in TMT A (Table 3), and small in TMT B and phonological verbal fluency task (Table 5(b)). We found slightly lower percentages of impaired RRMS patients in cognitive flexibility (28%), psychomotor speed (20%) and composite memory (16%) with significant differences compared to controls and small effect size (Tables 3, 5(b)). The cognitive domains that are mostly affected in our cognitively impaired RRMS patients are processing speed, demonstrated with significantly low reaction time and low scores in TMT A, followed by executive dysfunction, showen with poor performance in TMT B and phonological verbal fluency tasks.

SPMS patients performed significantly worse than control group in all tasks except complex attention. Higher percentages of cognitively impaired SPMS patients were found in reaction time (83.33%), TMT A and B (63.33% and 76.67% resp.), psychomotor speed and cognitive flexibility (66.67%), semantic and phonological verbal fluency tasks (53.33% and 50% resp.), and in composite memory (40%), all with large effect size except from semantic verbal fluency task that had medium effect size (Tables 3, 5(b)).

It appears that deficits in reaction time, TMT A and B, and phonological verbal fluency task are the hallmarks of cognitively impaired RRMS and SPMS patients followed by impaired cognitive flexibility, psychomotor speed, and composite memory. Our findings are consistent with previous studies highlighting that the most affected cognitive domains in MS are processing speed, executive function, and memory with relative preservation of language [11, 25].

Comparison between RRMS and SPMS patients demonstrated medium effect sizes on TMT B and psychomotor speed, while small effect sizes were present on composite memory and processing speed (Table 5(b)). Although composite memory, information processing, psychomotor speed, and TMT B deficits are present in RRMS patients, there is a significant decline in performance as disease progress, compatible with previous studies [26, 27].

In the present study, cognitive assessment was performed with CNS VS, a battery that uses computerized forms of traditional tests such as Symbol Digit Modalities and Stroop and can provide even nonneuropsychologist clinician with a reliable, highly sensitive screening tool for detecting cognitive deficits in MS patients. Computerized batteries have demonstrated comparable results to traditional neuropsychological batteries. They are easy to use, require significantly less time to administer, produce instant scoring and can incorporate alternate forms, necessary to minimize learning effect on follow-up. Computerized cognitive screening batteries have also the capacity to accurately-automatically quantify “speed factor” via multiple parameters such as reaction time, psychomotor speed, and processing speed, increasing their sensitivity in detecting even subtle changes in information processing speed [19, 28–33].

However, it is important to address few disadvantages of computerized cognitive assessment screening such as reliance on the visual modality and familiarity of the participant with computers. Moreover, there are many potential sources of error including use of various configurations and operating systems. There is also a provision of less qualitative information compared with paper and pencil tests [34, 35].

In order to broaden the neuropsychological assessment and try to designate differences in cognitive profile between RRMS and SPMS patients, we also administered four traditional tests TMT A and B semantic and phonological verbal fluency tasks. A face to face parallel testing by a traditional paper and pencil versus its computerized form will be of great importance to address the actual efficacy of computerized neuropsychological tests in detecting cognitive impairment.

In conclusion, the present study clearly demonstrated cognitive decline in RRMS and SPMS patients of a district Greek population in Western Greece, using a computerized battery (CNS VS) and traditional neuropsychological tests (TMT A and B semantic and phonological verbal fluency tasks). CNS VS appears to be sensitive in detecting cognitive impairment in RRMS and SPMS patients. Significant impairment occurs in almost all studied cognitive domains such as episodic memory, executive function, and processing speed with gradual increment of the frequency as disease progresses. We did not detect distinct patterns of impairment between MS subtypes. This suggests a relatively broad pattern of cognitive deficits in MS, independently of the disease course, but with gradual augmentation of the frequency as disease progresses.

## Figures and Tables

**Table 1:** Demographic and clinical characteristics of patients and controls.

	Controls	RRMS	SPMS	Significant differences
*N*	31	50	30	
Age (years)	40.742 (2.695)	41.760 (11.289)	48.800 (6.228)	RRMS < SPMS
Education (years)	12.935 (1.093)	12.260 (3.556)	12.667 (3.262)	n.s.
Gender M/F, %	38.7/61.3	22/78	26.7/73.3	n.s.
EDSS		3.050 (0.810)	6.183 (0.623)	RRMS < SPMS
Duration (years)		8.840 (4.053)	15.800 (5.135)	RRMS < SPMS
Hand, R/L %	90.2/9.8	94/6	93.3/6.7	n.s.

Values are mean (SD); *χ* ^2^ test for gender and hand, Kruskal-Wallis test for age, education, Mann-Whitney *U* test for EDSS, duration.

RRMS: relapsing remitting multiple sclerosis, SPMS: secondary progressive multiple sclerosis.

n.s: nonsignificant.

Significant differences: RRMS < SPMS with *P* < 0.001.

**Table 2:** Correlations between age, education, and all neuropsychological measures in our control group. Pearson's correlations for composite memory, executive function, and psychomotor speed. Spearman rank order correlations for all other measures.

	Age	Educ.	Tmta	Tmtb	Vfsem	Vfphon	Reactime	Compatt	Cognflex	Procsp	Compm	Psysp
Educ.	−0.11											
Tmta	−0.07	−0.14										
Tmtb	0.00	−0.10	0.72***									
Vfsem	−0.09	0.12	−0.44*	−0.59***								
Vfphon	0.03	0.05	−0.24	−0.42**	0.69***							
Reactime	0.14	−0.23	0.16	0.01	0.13	−0.03						
Compatt	0.04	−0.04	−0.02	0.30	−0.31	−0.30	−0.42*					
Cognflex	−0.03	0.18	−0.47**	−0.65***	0.62***	0.49***	−0.01	−0.58***				
Procsp	0.10	−0.21	−0.06	−0.11	−0.05	−0.37*	0.21	0.03	−0.09			
Compm	0.12	−0.02	−0.10	−0.33	0.08	0.30	−0.22	−0.25	0.40*	0.10		
Psysp	−0.11	0.02	−0.44*	−0.51**	0.32	0.12	−0.30	0.14	0.31	0.09	0.41*	
Exfunct	0.23	−0.03	−0.15	−0.09	−0.06	−0.23	0.12	0.00	−0.12	0.69***	0.05	−0.04

Correlation is significant at *P* < 0.001***, *P* < 0.01**, *P* < 0.05*.

Age; Educ.: education; tmta: trail making test a; tmtb: trail making test b; vfsem: verbal fluency semantic; vfphon: verbal fluency phonological; reactime: reaction time; compatt: complex attention; cognflex: cognitive flexibility; procsp: processing speed; compm: composite memory; psysp: psychomotor speed; exfunct: executive function.

**Table 3:** Frequency of impairment in each measure for each group of patients; number and percent %.

	RRMS (*N* = 50)	SPMS (*N* = 30)
Tmta	17 (34%)	19 (63.33%)
Tmtb	17 (34%)	23 (76.67%)
Vfsem	12 (24%)	16 (53.33%)
Vfphon	15 (30%)	15 (50%)
Compmem	8 (16%)	12 (40%)
Psyspeed	10 (20%)	20 (66.67%)
Reactime	29 (58%)	25 (83.33%)
Compatt	12 (24%)	11 (36.76%)
Cognflex	14 (28%)	20 (66.67%)
Procspeed	4 (8%)	8 (26.67%)
Exfunction	12 (24%)	7 (23.33%)

tmta: trail making test a; tmtb: trail making test b; vfsem: verbal fluency semantic; vfphon: verbal fluency phonological; compmem: composite memory; psyspeed: psychomotor speed; reactime: reaction time; compatt: complex attention; cognflex: cognitive flexibility; procspeed: processing speed; exfunction: executive function.

RRMS: relapsing remitting multiple sclerosis; SPMS: secondary progressive multiple sclerosis.

**Table:** (a)

	Age	Education	EDSS	Duration
Education	−0.35*			
EDSS	0.28	0.11		
Duration	0.58***	−0.10	0.58***	
Tmta	0.31*	−0.44**	0.14	0.24
Tmtb	0.43**	−0.41**	0.21	0.30*
Vfsem	−0.44**	0.42**	−0.09	−0.20
Vfphon	−0.41**	0.46***	−0.11	−0.23
Reactime	0.37**	−0.32*	0.16	0.36**
Compatt	0.23	−0.38**	0.05	0.04
Cognflex	−0.30*	0.40**	−0.13	−0.19
Procspeed	−0.60***	0.47***	−0.31*	−0.43**
Compmem	−0.44**	0.30*	−0.29*	−0.39**
Psyspeed	−0.44**	0.33*	−0.40**	−0.40**
Exfunction	−0.34*	0.44**	−0.13	−0.20

Correlation is significant at *P* < 0.001***, *P* < 0.01**, *P* < 0.05*.

tmta: trail making test a; tmtb: trail making test b; vfsem: verbal fluency semantic; vfphon: verbal fluency phonological; reactime: reaction time; compatt: complex attention; cognflex: cognitive flexibility; procspeed: processing speed; compmem: composite memory; psyspeed: psychomotor speed exfunction: executive function.

**Table:** (b)

	Age	Education	EDSS	Duration
Education	0.23			
EDSS	0.07	0.05		
Duration	0.30	−0.11	0.35	
Tmta	0.31	−0.28	0.03	0.17
Tmtb	0.00	−0.17	−0.11	−0.07
Vfsem	0.05	0.25	0.25	0.13
Vfphon	0.13	0.37*	0.10	0.09
Reactime	−0.13	−0.07	−0.22	−0.31
Compatt	0.20	−0.43*	−0.08	0.37*
Cognflex	0.00	0.10	0.15	−0.12
Procspeed	0.02	0.04	0.00	0.04
Compmem	0.10	0.07	−0.32	0.14
Psyspeed	−0.32	0.17	−0.28	−0.26
Exfunction	−0.23	0.13	0.04	−0.17

Correlation is significant at *P* < 0.001***, *P* < 0.01**, *P* < 0.05*.

tmta: trail making test a; tmtb: trail making test b; vfsem: verbal fluency semantic; vfphon: verbal fluency phonological; reactime: reaction time; compatt: complex attention; cognflex: cognitive flexibility; procspeed: processing speed; compmem: composite memory; psyspeed: psychomotor speed exfunction: executive function.

**Table:** (a)

	RRMS	SPMS	Controls
Tmta	51.894 (19.549)	63.436 (16.527)	37.645 (12.547)
Tmtb	97.543 (39.400)	141.537 (41.787)	79.646 (22.946)
Vfsem	43.240 (10.413)	36.967 (10.950)	47.419 (8.865)
Vfphon	29.340 (12.327)	21.800 (9.393)	34.871 (9.552)
Compmem	90.940 (9.851)	83.300 (9.675)	97.903 (11.915)
Psyspeed	135.520 (26.301)	103.267 (27.875)	148.387 (24.204)
Reactime	827.360 (185.026)	960.433 (143.748)	618.516 (93.790)
Compatt	15.180 (10.435)	19.733 (9.755)	13.129 (6.820)
Cognflex	26.400 (22.707)	14.200 (13.387)	37.968 (15.070)
Procspeed	39.580 (15.617)	26.800 (10.387)	40.613 (14.986)
Exfunction	26.940 (22.835)	15.733 (13.460)	33.484 (16.442)

RRMS: relapsing-remitting multiple sclerosis; SPMS: secondary progressive multiple sclerosis; tmta: trail making test a; tmtb: trail making test b; vfsem: verbal fluency semantic; vfphon: verbal fluency phonological; compmem: composite memory; psyspeed: psychomotor speed; reactime: reaction time; compatt: complex attention; cognflex: cognitive flexibility; procspeed: processing speed; exfunction: executive function.

**Table:** (b)

	RRMS versus SPMS	RRMS versus Controls	SPMS versus Controls
Tmta	Hedges' *g* = 0.345	Hedges' *g* = −0.455**	Hedges' *g* = −0.672***
	*P*-value = 0.071	*P*-value = 0.001	*P*-value = 0.000

Tmtb	Hedges' *g* = 0.770**	Hedges' *g* = −0.487*	Hedges' *g* = −1.313***
	*P*-value = 0.002	*P*-value = 0.020	*P*-value = 0.000

Vfsem	Hedges' *g* = −0.280	Hedges' *g* = 0.404	Hedges' *g* = 0.758**
	*P*-value = 0.060	*P*-value = 0.074	*P*-value = 0.002

Vfphon	Hedges' *g* = −0.391	Hedges' *g* = 0.432*	Hedges' *g* = 0.979***
	*P*-value = 0.085	*P*-value = 0.027	*P*-value = 0.000

Compmem	Hedges' *g* = −0.483*	Hedges' *g* = 0.627*	Hedges' *g* = 0.978***
	*P*-value = 0.046	*P*-value = 0.013	*P*-value = 0.000

Psyspeed	Hedges' *g* = −0.804**	Hedges' *g* = 0.455*	Hedges' *g* = 1.226***
	*P*-value = 0.001	*P*-value = 0.049	*P*-value = 0.000

Reactime	Hedges' *g* = 0.421	Hedges' *g* = −1.347***	Hedges' *g* = −2.052***
	*P*-value = 0.065	*P*-value = 0.000	*P*-value = 0.000

Compatt	Hedges' *g* = 0.227	Hedges' *g* = −0.096	Hedges' *g* = −0.268
	*P*-value = 0.319	*P*-value = 0.550	*P*-value = 0.106

Cognflex	Hedges' *g* = −0.328	Hedges' *g* = 0.554*	Hedges' *g* = 1.122***
	*P*-value = 0.120	*P*-value = 0.010	*P*-value = 0.000

Procspeed	Hedges' *g* = −0.565*	Hedges' *g* = 0.013	Hedges' *g* = 0.513*
	*P*-value = 0.014	*P*-value = 0.957	*P*-value = 0.046

Exfunction	Hedges' *g* = −0.286	Hedges' *g* = 0.287	Hedges' *g* = 0.708**
	*P*-value = 0.169	*P*-value = 0.187	*P*-value = 0.007

*P* < 0.001***, *P* < 0.01**, *P* < 0.05*.

RRMS: relapsing remitting multiple sclerosis; SPMS: secondary progressive multiple sclerosis; CONTR: controls; tmta: trail making test a; tmtb: trail making test b; vfsem: verbal fluency semantic; vfphon: verbal fluency phonological; compmem: composite memory; psyspeed: psychomotor speed; reactime: reaction time; compatt: complex attention; cognflex: cognitive flexibility; procspeed: processing speed; exfunction: executive function.
